# Supplementary material for: Mapping the evolution of fertility support policies in China: A content and instrumental analysis
Source: PLoS One. 2025 Oct 9;20(10):e0332137. doi: 10.1371/journal.pone.0332137 (PMC12510515; doi:10.1371/journal.pone.0332137)
Supplement: S1 Appendix — (ZIP) [file pone.0332137.s001.zip › S1 Appendix. 226 original policy documents/109-人口计生委等14部门关于全面加强农村人口和计划生育工作的若干意见(FBM-CLI-4-99532).doc]

人口计生委等14部门关于全面加强农村人口和计划生育工作的若干意见

**发布部门：** [国家人口和计划生育委员会(含国家计划生育委员会)(已撤销)](https://www.pkulaw.com/law/chl?Aggs.IssueDepartment=60304) [机构沿革](javascript:void(0))

**发布日期：**2007.11.08

**实施日期：**2007.11.08

**时效性：** [现行有效](https://www.pkulaw.com/law/chl?Aggs.TimelinessDic=01)

**效力级别：** [部门规范性文件](https://www.pkulaw.com/law/chl?Aggs.EffectivenessDic=XE0302)

**法规类别：** [计划生育管理](https://www.pkulaw.com/law/chl?Aggs.Category=09705)

人口计生委等14部门关于全面加强农村人口和计划生育工作的若干意见

各省、自治区、直辖市人口计生委、发展改革委、教育厅（教委）、公安厅（局）、民政厅（局）、财政厅（局）、人事厅（局）、劳动保障厅（局）、国土资源厅（局）、建设厅（建委）、农业厅（局、委、办）、卫生厅（局）、农村综改办、扶贫办，新疆生产建设兵团人口计生委、发展改革委、教育局、公安局、民政局、财务局、人事局、劳动保障局、国土资源局、建设局、农业局、卫生局、农村综改办、扶贫办：
　　为深入贯彻党的十七大精神以及《[中共中央国务院关于全面加强人口和计划生育工作统筹解决人口问题的决定](https://www.pkulaw.com/chl/5e7b01010a4102ffbdfb.html)》（中发[2006]22号，以下简称中央《决定》），全面加强农村人口和计划生育工作，现提出如下意见：
　　一、充分认识全面加强农村人口和计划生育工作的必要性和紧迫性
　　农村人口问题是解决"三农"问题和建设社会主义新农村的关键因素。30多年来，农村人口和计划生育工作取得了巨大成就，广大农民为农村进入低生育水平和全国低生育水平的稳定作出了突出贡献。同时，必须清醒地看到，当前农村人口和计划生育工作面临着前所未有的挑战。我国农村人口比例大、社会经济发展相对滞后、社会保障制度不健全、传统婚育观念尚未根本转变、人口流动日趋频繁，一些农村计划生育家庭仍面临生产、生活和养老等方面的实际困难，稳定农村低生育水平的任务十分艰巨。人口和计划生育工作仍然是"天下第一难事"，重点和难点依然在农村。
　　全面落实科学发展观，构建社会主义和谐社会迫切需要统筹解决人口数量、素质、结构和分布等问题；统筹城乡发展，推进社会主义新农村建设，对人口和计划生育工作提出了新的更高的要求。为此，要牢牢把握农村人口和计划生育工作的基础性地位和阶段性特征，坚持农村经济社会发展与人口和计划生育工作协调推进，坚持国家指导与群众自愿相结合的原则，坚持把帮助解决群众实行计划生育的现实困难摆在首位，把实现好、维护好、发展好广大农民的根本利益作为一切工作的出发点和落脚点，加快转变工作思路和工作方法。
　　新时期农村人口和计划生育工作总的要求是：以党的十七大精神为指导，深入贯彻落实中央《决定》，将农村人口和计划生育工作纳入社会主义新农村建设的总体部署，完善利益导向政策，强化服务和管理体系，建立健全依法管理、村民自治、优质服务、政策推动、综合治理的长效工作机制，维护群众实行计划生育的合法权益，引导群众自觉实行计划生育，稳定低生育水平，统筹解决人口问题，为促进建设社会主义新农村、构建社会主义和谐社会、全面建设小康社会创造良好的人口环境。

　　二、积极推进新农村新家庭计划
　　新农村新家庭计划旨在依托人口和计划生育网络，倡导和促进健康文明的行为和生活方式，保障农民享有基本的生殖健康服务，培育一大批"富裕文明、身心健康、优生优育、权益保障、民主和谐"的新家庭，稳定低生育水平，统筹解决人口问题，促进社会主义新农村建设。
　　实行计划生育，提高农民家庭生活水平是新农村新家庭计划的根本目标。要研究制定新农村新家庭的评估标准，把群众实行计划生育作为一项基本要求。组织群众参与新农村新家庭计划，把群众实行计划生育和脱贫致富结合起来，利用人口学校、计划生育服务机构、人口文化大院等阵地，发挥人口和计划生育网络、信息、组织优势以及各相关部门的优势，为计划生育家庭提供优先优惠的生产、生活以及生育等公共服务。
　　大力提高出生人口素质和农村文明健康水平是新农村新家庭计划的主要任务。各有关部门要各司其职、密切配合，全面开展出生缺陷预防，重点推广一级预防措施，加强婚育咨询指导及孕前保健，推广使用预防出生缺陷的新产品新技术；积极实施二级预防措施，全面开展产前诊断工作，提高孕期出生缺陷发现率；努力推进三级预防措施，开展新生儿和婴幼儿系统保健，提高新生儿疾病筛查率。实施计划生育生殖健康促进计划，做好孕前管理和服务，引导群众知情选择避孕节育措施。深入开展计划生育优质服务先进单位创建活动。借鉴国际先进理念，积极开展国际合作项目，发挥项目地区的示范作用。开展婴幼儿早期教育，强化独生子女社会行为教育和培养。
　　加强农村社区新型生育文化建设是新农村新家庭计划的重要内容。要深入开展婚育新风进万家活动和关爱女孩行动，综合治理出生人口性别比偏高问题。加强农村人口文化阵地建设。广泛开展人口国情、人口和计划生育法律法规及生育政策的宣传教育。立足农村社区实际，倡导树立男女平等、少生优生等文明婚育观念，倡导女孩参与社区文化活动，鼓励男到女家落户，逐步改变重男轻女等封建传统意识和习俗，逐步消除性别歧视。
　　新农村新家庭计划是社会主义新农村建设的一项重要基础工程。新农村建设试点应有新家庭计划的内容，研究制定相关经济社会政策要有利于人口和计划生育工作。要把实施新农村新家庭计划纳入到社会主义新农村建设总体规划之中，根据各地的人口形势以及工作基础，选择不同经济发展类型的村或乡镇开展试点。积极争取地方各级政府加强对试点工作的指导，并提供必要的保障。各相关部门要互相协调、积极配合，制定具体政策措施，将新家庭计划和新农村建设的各项工作同部署、同落实、同检查。

　　三、建立和完善人口和计划生育利益导向政策体系
　　人口和计划生育利益导向政策是惠民政策的重要组成部分，是国家指导群众实行计划生育的重要手段。计划生育家庭为国家作出贡献，应当优先分享改革发展的成果，困难家庭应当得到重点扶助。建立健全利益导向基本制度。
　　要切实落实法律法规规定的各项计划生育奖励优待政策。全面实施农村部分计划生育家庭奖励扶助制度和"少生快富"工程，落实独生子女父母奖励和计划生育免费基本技术服务制度。加快建立计划生育家庭特别扶助制度，探索建立长效节育措施奖励、节育手术保险等制度。完善有奖举报制度，建立健全相关奖惩政策措施。对违法生育的，依法征收社会抚养费，严格执行"收支两条线"的管理规定；是党员、干部的，依法依纪从严惩处。
　　做好惠民政策与计划生育奖励优待政策的衔接。要按照《[土地管理法](https://www.pkulaw.com/chl/098c17d5cfc8b2a7bdfb.html)》的有关规定，以征地面积为主要依据计算和分配土地补偿费和安置补助费，并对独生子女户、双女户予以照顾。移民搬迁、危旧房改造等补助政策，要以家庭作为基本的补助单位，并适当提高对计划生育纯女户危旧房改造补助标准。享受计划生育奖励优待政策的群众，不影响其同时享受其他惠民政策待遇。按照国家规定所获得的计划生育奖励与扶助金，在开展农村最低生活保障时一般不计入家庭收入。符合农村五保供养条件的独生子女死亡家庭，应落实农村五保供养待遇，并按规定享受计划生育扶助。
　　制定和落实惠民政策要体现对计划生育家庭的优先优惠。农村扶贫开发、宅基地划分、改水改厕、沼气应用、新技术推广等政策或项目，要对农村独生子女户和双女户给予优待和照顾。要将独生子女和双女父母优先纳入劳动力转移"阳光工程"、"雨露工程"培训之中。将计划生育纯女户作为各级各部门结对帮扶的重点对象，推广"幸福工程"等社会公益活动。

　　四、探索建立农村计划生育家庭养老保障制度
　　随着农村人口老龄化程度不断加深，计划生育家庭的养老问题日益突出。要从农村计划生育家庭，特别是独生子女户和双女户着手，探索建立农村养老保险制度，创新养老模式，逐步解除农民实行计划生育的养老之忧。落实五保供养制度，解决好"三无"老人的保障问题。继续发挥土地养老的保障作用，巩固家庭养老保障的基本功能。
　　要与农村经济发展水平相适应，与其他保障措施相配套，调动政府、集体和个人的积极性，多方筹集资金，探索建立多种形式的农村计划生育家庭养老保险制度。鼓励各地为独生子女户和双女户因地制宜地开展养老保障项目。探索开展农村养老保险的地区要优先考虑独生子女户和双女户。
　　积极开展助老养老服务。鼓励和调动社会力量，采取公建民营、民办公助等多种形式，加快农村养老机构建设，不断满足农村空巢老人和留守老人的生活照料需求，优先和优惠为身边无子女的计划生育老人提供集中供养服务。对生活不能自理的农村计划生育家庭老年父母，按规定提供适当补助。要大力弘扬中华孝道、子女赡养、邻里互助的风尚，开展爱老助老义工活动，倡导社会各界关爱农村老人。

　　五、不断完善流动人口计划生育服务和管理
　　流动人口的主体是农民工。各地要按照中央《决定》和《[国务院关于解决农民工问题的若干意见](https://www.pkulaw.com/chl/de43d86a655f08cfbdfb.html)》（国发[2006]5号）的要求，坚持以流入地为主、流出地和流入地协调配合的工作机制，按照统一管理、优质服务的要求，积极探索不同类型的区域协作模式，创新管理方式和服务手段，推动形成党政领导统筹协调、各部门密切配合、流入地和流出地互动互补的"一盘棋"格局，统筹解决好流动人口问题。
　　流出地要从源头上做好流动人口计划生育服务和管理工作。流出地要做好外出人员的宣传、教育、培训，落实避孕节育措施，免费办理《流动人口婚育证明》。配合流入地，及时提供、反馈流出人口相关信息。依法落实流出人口及其家庭应该享有的奖励优惠政策。建立定期联系制度，加强对流动人口，特别是农民工家庭的上门访视工作，帮助计划生育留守家庭及子女解决实际困难。
　　流入地要按照"属地化管理、市民化服务"的原则，将流动人口纳入当地人口总数，将流动人口计划生育纳入流动人口服务和管理工作的全局，把相关部门配合开展流动人口计划生育工作的职责落实情况纳入目标管理责任范围，落实流动人口计划生育服务和管理经费。流动人口可以凭《流动人口婚育证明》享有国家政策规定的计划生育、生殖健康基本项目免费服务，逐步做到与户籍人口享有同等的服务。完善企事业单位法定代表人计划生育工作责任制，加强城市社区流动人口信息登记通报制度，将流动人口计划生育服务管理列入流动人口协管员工作职责。在流动人口集中的社区、企业、集贸市场等成立流动人口计划生育协会，广泛调动社会力量做好流动人口计划生育工作。
　　加强信息化建设和考核评估工作。利用政府及各部门信息网络和资源，完善流动育龄妇女信息系统。完善全国流动人口计划生育信息交换平台，开展跨省或省内流动人口计划生育信息交换与共享试点。建立科学合理的流动人口计划生育工作评估标准，研究建立目标管理双向考核机制。

　　六、进一步强化人口和计划生育基层基础工作
　　人口和计划生育工作是农村社会管理和公共服务的重要内容。落实计划生育基本国策关键靠农村基层组织，靠基础工作。要适应农村基层政权建设和综合改革的新形势，强基固本，确保人口和计划生育工作机构和人员稳定。要按照辖区常住人口规模和工作需要，科学设立行政管理、技术服务机构，合理配备工作人员，不得随意撤并和改变机构性质。加强县乡计划生育服务标准化和规范化建设，改善计划生育服务机构设施和装备水平。加强人口和计划生育队伍职业化建设，强化教育培训，提高整体素质。县乡两级人口和计划生育工作岗位特殊、任务艰巨，要按规定落实基层工作人员的工资和社会保障待遇。
　　把人口和计划生育工作纳入农村社区基层管理服务体系。建立健全村级计划生育专干队伍，确保人员、任务和待遇"三落实"。村级计划生育专干一般要在35周岁以下、初中以上文化程度、以女性为主，实行"县管、乡聘、村用"，鼓励民主选举、民主评议村级计划生育专干。适当提高计划生育专干待遇，其报酬可参照村委会主要干部的标准。
　　深入开展计划生育村民自治。推进基层人口和计划生育行政管理体制改革，加快职能转变，加强对村级计划生育工作的分类指导和支持力度。积极创造条件，维护群众实行计划生育的知情权、选择权和生殖健康权。全面推行计划生育村务公开，依法制定包括计划生育内容的村民自治章程或村规民约，引导村级集体收益和资源分配有利于实行计划生育的个人和家庭。进一步发挥计划生育协会的作用，维护群众权益，实现群众自我管理、自我服务、自我教育和自我监督。
　　积极推进基层人口和计划生育工作信息化。抓好人口宏观管理与决策信息系统项目建设，完善育龄妇女信息系统以及各相关管理服务信息系统，发挥信息引导和支持作用。建立健全出生医学证明、户籍管理、婚姻登记等公共管理服务信息系统，建立各级部门间人口信息共享机制和方式，在保护个人隐私的前提下，逐步实现与人口和计划生育管理服务信息系统的互联互通和业务协同。未建立上述信息管理系统的，应根据需要采取多种方式建立各级部门间定期信息采集制度。

　　七、切实加强对农村人口和计划生育工作的领导
　　各地各有关部门要深刻认识农村人口和计划生育工作的重要性、长期性、艰巨性和复杂性，把人口发展放在与经济建设同等重要的位置，一手抓经济建设，一手抓人口发展，做到人口和计划生育工作与经济工作同安排、同落实。要充分发挥各级人口和计划生育工作领导小组的作用，建立和完善部门协调会议制度，定期研究解决重大问题，协调制定相关社会经济发展政策。各相关部门在制定完善惠民政策时要充分考虑计划生育基本国策，积极开展对人口发展影响的评估，主动征求人口和计划生育部门的意见。探索建立人口发展指标评估体系，改革和完善人口和计划生育目标管理责任制。
　　切实加强对农村人口和计划生育工作的投入。建立稳定增长的投入保障机制。人口和计划生育投入增长幅度要高于经常性财政收入增长幅度，确保法律规定的各项奖励优惠政策、县乡人口和计划生育技术服务机构基本建设和队伍建设、计划生育经常性工作、计划生育免费基本技术服务等经费的落实。强化对财政资金到位情况和资金使用绩效的考核，加强监督检查，提高资金使用效益。
　　坚决推进依法行政。积极创造依法行政的环境和条件，将稳定低生育水平、完成人口计划与依法行政、维护群众合法权益、帮助解决群众实际困难有机结合起来。加强法制宣传教育和培训，提高执法人员的执法水平和群众的守法意识。全面落实行政执法责任制。及时了解群众诉求，把矛盾化解在基层、化解在萌芽状态，杜绝恶性案件和群体性事件的发生。对出现重大违法侵权案件和恶性案件的，坚决实行"一票否决"。
　　把建立健全依法管理、村民自治、优质服务、政策推动、综合治理的长效工作机制，作为确保农村人口和计划生育工作持续稳定健康发展的根本措施，大力推进理论创新、制度创新、能力创新和载体创新，与其他各项改革和建设有机结合，统筹兼顾、协调推进，使之与当地经济社会发展水平相协调，与人民群众需求相吻合。加快建立完善长效工作机制建设的考核评估体系，力争到2010年全国大多数地区基本建立长效工作机制。
　　各地要根据本地人口和计划生育工作实际，结合本意见，制定具体的实施意见。请各地将贯彻落实本意见的情况以及存在的问题，及时报告给国家各有关部门。

人口计生委
发展改革委
教　育　部
公　安　部
民　政　部
财　政　部
人　事　部
劳动保障部
国土资源部
建　设　部
农　业　部
卫　生　部
国务院农村综合改革工作小组
国务院扶贫办
二00七年十一月八日

© 北大法宝：（[www.pkulaw.com](https://www.pkulaw.com/)) 专业提供法律信息、法学知识和法律软件领域各类解决方案。北大法宝为您提供丰富的参考资料，正式引用法规条文时请与标准文本核对。
欢迎查看所有[产品和服务](https://www.pkulaw.com/helps/2.html)。法宝快讯：[如何快速找到您需要的检索结果？](https://www.pkulaw.com/helps/70.html)[法宝](https://www.pkulaw.com/helps/69.html)V6有何新特色？

扫描二维码阅读原文

**原文链接：**<https://www.pkulaw.com/chl/89bad8c5d20e6d2fbdfb.html>
